# Thermal imaging of soybean response to drought stress: the effect of *Ascophyllum nodosum* seaweed extract

**DOI:** 10.1186/s40064-016-3019-2

**Published:** 2016-08-22

**Authors:** Alex Martynenko, Katy Shotton, Tessema Astatkie, Gerry Petrash, Christopher Fowler, Will Neily, Alan T. Critchley

**Affiliations:** 1 Faculty of Agriculture, Dalhousie University, Truro, NS B2N 5E3 Canada; 2 Acadian Seaplants Limited, 30 Brown Ave., Dartmouth, NS B3B 1X8 Canada

**Keywords:** Leaf temperature, Leaf angle, Turgor, Stomatal conductance, Plant stress, Water deficit, Seaweed extract

## Abstract

Previous experiments have demonstrated positive effect of Acadian^®^ extract of *Ascophyllum nodosum* on plant stress-resistance, however the mode of action is not fully understood. The aim of this study was to understand the physiological effect of Acadian^®^ seaweed extract on the plant response to drought stress. Leaf temperature and leaf angle were measured as early-stage indicators of plant stress with thermal imaging “in situ” over a 5-day stress-recovery trial. The early stress-response of control became visible on the third day as a rapid wilting of leaves, accompanied with the asymptotic increase of leaf temperature on 4–5 °C to the thermal equilibrium with ambient air temperature. At the same time Acadian^®^ treated plants still maintained turgor, accompanied with the linear increase in leaf temperature, which indicated better control of stomatal closure. Re-watering on the fifth day showed better survival of treated plants compared to control. This study demonstrated the ability of Acadian^®^ seaweed extract to improve resistance of soybean plants to water stress.

## Background

Soybean is ranked among the most important agricultural food crops, being an essential part of food security (Akibode and Maredia 2011). Production of pulses, groundnuts and soybean have followed an increasing trend with the global production tripling from 148 million tons in 1980–1982 to 480 million tons in 2012–2014. The increase was led by soybean production which increased from 87 million tons to 214 million tons due to an increase in the demand for protein meals and oils. Such trends push soybean agriculture in to marginal semi-arid areas, where water-limiting conditions often constrain crop productivity (Darianto et al. 2015).

Soybean plants are very sensitive to drought stress, especially during reproduction. According to Iowa State data (Lenssen 2012), 4 days of visible moisture stress in the 3rd week of pod development results in about 36 % loss, increasing to 39–45 % in the 2nd–4th week of seed filling. Drought stress causes abortion of small pods, reduced seeds per pod, and reduced seed size. Currently, the economically viable approaches to support crop production under drought are still limited. Further research of physiological effects of natural extracts as a possible tool to improve plant resistance to abiotic stresses could be a viable strategy.

It is well-known that seaweed extracts improve the stress tolerance of agricultural crops. The unique properties and diverse functionality of seaweed extracts for agricultural applications have been extensively reviewed (Craigie 2011; Arioli et al. 2015). Numerous studies have shown the positive effect of Acadian^®^ extract of *Ascophyllum nodosum* (Acadian Seaplants, Nova Scotia, Canada) on plant resistance to drought stress (Neily et al. 2010; Spann and Little 2011). However, the modes of action of seaweed extract to improve stress tolerance are not fully understood.

The response of higher plants to drought stress is a complex and dynamic process (Chaves et al. 2003). The plant leaf is a primary receptor of stress, triggering a chain of physiological responses from gene expression and hormone regulation (Peleg and Blumwald 2011) to osmoregulation (Ahmad and Wani 2014) and structural adaptation (Bacelar et al. 2004). The plant leaf has an inherent mechanism of stomatal regulation, which controls gaseous exchange, photosynthesis and metabolic activities in response to environmental changes by maintaining a crucial balance between photosynthetic gains and water losses (Chaves et al. 2003; Jones et al. 2009; Costa et al. 2013).

Abscisic acid (ABA)-mediated stomata closure is one of the first plant responses to drought stress (Hetherington and Woodward 2003). Although guard cells can lose turgor as a result of a direct loss of water, stomatal closure in response to dehydration is always an active, energy-dependent process (Hetherington and Woodward 2003). Stomatal closure results in reduction of stomatal conductance and CO_2_ availability, which directly affects rates of photosynthesis (Chaves et al. 2003). It is accompanied by an increase in leaf temperature (Jones 1999a). If this temperature reaches a threshold, it often leads to irreversible leaf tissue damage. Hence, leaf temperature can be used as an indicator of plant stress (Jones 1999a; Jones et al. 2009; Costa et al. 2013). Crop water stress index (CWSI) is calculated, using equation (Idso et al. 1981):

1 $$CWSI = \left( {T_{canopy} {-}T_{nws} } \right)/\left( {T_{{max} } {-}T_{nws} } \right)$$

where *T*_*max*_ is a temperature of the dry leaf surface and *T*_*nws*_ is the temperature under non-limiting soil water condition, when crop transpiration is at its maximum rate. Fuentes et al. (2012) developed automated methodology for measurements of water stress index of grapevine canopies, using thermal imaging. However, field application of this equation encountered several problems, including difficulty in separation of relevant crop canopy temperature from background and normalization of CWSI under changing climatic conditions. For practical purposes, Jones proposed simplified “index of relative stomatal conductance” *I*_*g*_, which assumes constant environmental conditions (Jones 2004):

2 $$I_{g} = (T_{dry} - T_{leaf} )/(T_{leaf} - T_{wet} ) = g_{s} /G$$

where *g*_*s*_ is stomatal conductance (m/s), *G* is the constant, calculated as a slope of *g*_*s*_(*I*_*g*_) regression. The advantage of using thermal index is linear relationship between leaf temperature and stomatal conductance (Jones 1999a, b). It is important to note that (2) can be used to evaluate stomatal conductance in controlled environment, where leaf temperature is mostly dependent on water availability. However, field applications of this equation are limited because of the side effects of environmental factors, such as air temperature, light intensity, relative humidity, wind speed and soil conditions (Jones and Schofield 2008; Leinonen et al. 2006).

Thermal imaging was successfully used to measure temperature of single leaves (Chaerle and Van Der Straeten 2000; Ribeiro da Luz and Crowley 2007; Kapanigowda et al. 2013) and whole plant (Grant et al. 2007; Blonquist et al. 2009; Zia et al. 2013; Fuentes et al. 2012). Theory and applications of thermal imaging to the study of plant water relations was reported by Jones (2004). The theoretical background of thermal imaging, in particular the relationship between leaf temperature and stomatal conductance, was thoroughly reviewed by Cohen et al. (2005) and Maes and Steppe (2012). An excellent review of digital image processing for detecting, quantifying and classifying plant diseases was presented by Barbedo (2013). Although imaging-guided expert systems are widely used in medicine (Egger 2013) and agriculture (Ishimwe et al. 2014), there is a gap in applications of real-time thermal imaging in plant physiology. As to our knowledge, thermal imaging has not been applied to study the stress-response of plants treated with biostimulants, such as seaweed extracts.

The initial hypothesis was that thermal imaging is able to detect early-stage physiological response of soybean plants to water stress “in-situ”. To test this hypothesis, the temperature and leaf angle of individual plants were measured over a 5-day stress-recovery experiment. In order to understand the mode of action of Acadian^®^ seaweed extract, responses of treated and untreated control plants in the same stress conditions were compared. It was anticipated that imaging of early-stage plant stress response would significantly advance our knowledge about the mode of action of seaweed extracts. This research is an important step towards understanding the full benefits of seaweed extracts for improving crop yield under water stress.

## Methods

### Plant preparation

Soybean seeds (*Glycine max* (L.) Merr) variety Savana were planted in ProMix BX (Premier Tech Horticulture, Canada) in 200-seed trays, in a controlled environment room (27 °C 16:8 day/night). After 7 days, the plants were transplanted into 4.5 inch pots and placed in a Conviron environmental chamber (Winnipeg, Canada) with fluorescent lamps Sylvania Hg Pentron 4100 K, 39 W (Osram Sylvania, USA), After an additional 14 days, drought stress was initiated. The environmental chamber was set at 16:8 day/night with the temperature 27 °C, ~400 µmol/m^2^/s PAR intensity, 600 ppm CO_2_ and 60 % relative humidity. Due to additional heat being generated by the lights, the temperature reached 33 °C during the day. Twice a week the plants were treated with 100 mL solution of 0.5 g/L 20-8-20 (Plant Products, Canada) or 0.5 g/L 20-8-20 plus 7.0 mL/L Acadian^®^. On Day 21, plants were treated with 1.0 g/L 20-8-20 or 1.0 g/L 20-8-20 plus 7.0 mL/L Acadian^®^ until the soil was completely saturated and the excess liquid ran through the bottom of the pots (~300 mL). This equalised the soil moisture content at ~70 % for the onset of drought stress and no further water was applied. Each experiment consisted of two plants (one treated, one control). Three separate experiments were conducted using a total of six plants (three treated, three control). A 5-day stress-recovery experiment with re-watering on the fifth day allowed observation of all stages of drought stress, i.e., stress response (Day 3), adaptation (Day 4) and recovery (Day 5). Data for leaf temperatures and angles were recorded from one pot as a biological replicate (two pots per experiment). Statistics were conducted on results from three experiments.

### Thermal imaging

A T440 IR camera (FLIR Systems Inc., North Billerica, MA, USA) with the focal plane array (FPA) uncooled microbolometer, 320 × 240 pixels and spectral range of 7.5–13 µm was used to take both thermal and RGB visible images automatically every 10 min. Camera settings for emissivity was constant 0.95 for the entire experiment. The imaging camera was installed in a Conviron environmental chamber at a distance of 1.1 m from the plants. In order to minimize the effects of wall reflectance and air temperature gradients due to vertical convective airflow, plants were shielded with 1.0×1.0 m Styrofoam sheets. This measure provided 0.1 °C temperature resolution in the field of view. One treated and one control plant were monitored in each experiment. Leaf temperature data and background temperature were measured in the following two modes:

1. *Online (camera) measurements*: Two rectangular regions of interest (ROI) were set directly on the camera screen. Thermal images displayed maximum and minimum temperatures within the ROI for each image in continuous mode of operation. Before stress the minimum temperature corresponded to leaf temperature, while the maximum temperature reflected ambient air temperature. After stress the minimum temperature represented air temperature, while the maximum temperature represented leaf (or stem) temperature.
2. *Offline measurements*: The data were recorded in form of radiometric images and analysed, using FLIR Research IR 4.1 software (FLIR Systems Inc., North Billerica, MA, USA).

**Fig. 1:**
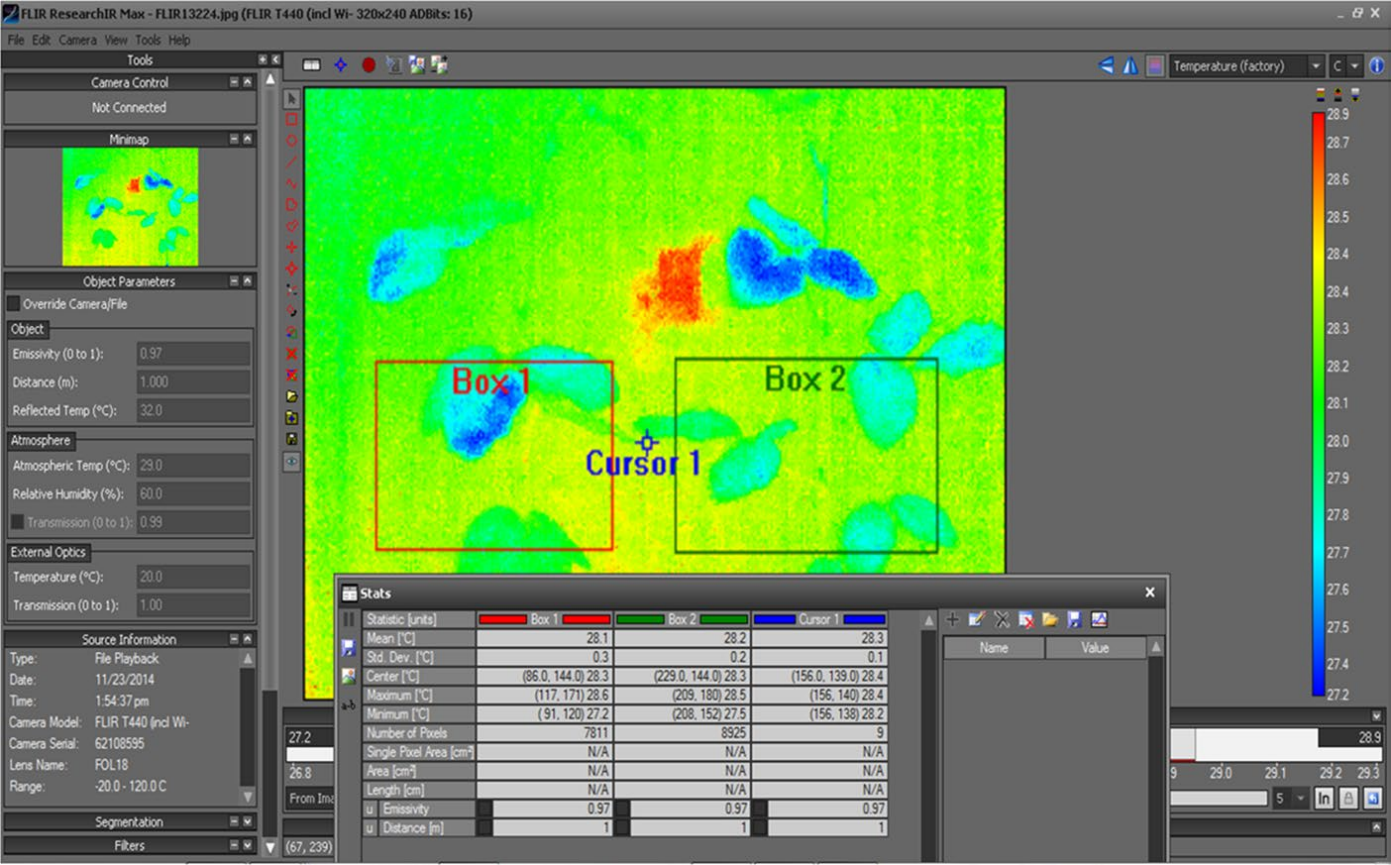
Screenshot of FLIR Research IR 4.1 software (FLIR Systems Inc., USA) for thermal image analysis

The software offered computation of maximum and minimum temperatures from any pre-defined ROI by using rectangle (Fig. 1), oval or user-defined shape selection. Figure 1 shows statistical data, which represent leaf temperature distribution for each plant. The air temperature was recorded from a ROI measurement cursor (3 × 3 pixels) at the point between the two plants. This technique allowed closer examination of the leaf temperatures and adjustment of the ROI if one plant grew into the ROI for the other plant. Also, as the apical point wilted, it would occasionally bend down into the ROI and interfere with the maximum temperature results. In this case the leaf temperature was corrected, using Research IR software. Online automated measurements were used as a primary source of information, whereas offline manual measurements were used to double-check measured values.

### Leaf angle and turgor calculations

Data for the leaf angle were obtained from the radiometric image by segmentation and using a digital protractor (IMAQ Vision Assistant, USA) with the vertical axis aligned to the stem. Leaf turgor was calculated from the leaf angle assuming a linear relationship. An angle of 90°, observed for horizontally oriented leaves, was associated with full turgor (100 %). An angle of 0°, observed for wilted plants, was interpreted as zero turgor (0 %). The thermal images collected allowed for measurements of leaf angle not only during the daytime, but also during some of the night periods.

### Statistical analysis

Two statistical methods, namely “repeated measures analysis” and “non-linear regression modeling”, were applied to the data sets (Montgomery 2013). Repeated measures analysis was completed to determine the effect of the Acadian^®^ treatment on leaf temperature and turgor, and how this changed over time. The experimental design adopted was the Randomized Block Design, with three blocks (i.e., three different experiments), two treatments (i.e., Acadian^®^-treated vs. control), and the temperature and turgor responses averaged for every hour during 10 h of daytime period. Hourly estimates of responses were taken as a mean value of data from six images. Akaike Information Criterion (Littell et al. 1998) was used to determine the most appropriate co-variance structure (compound symmetry) for both responses. The analysis was completed using the Mixed Procedure of SAS (SAS Institute Inc. 2014), and further multiple means comparison was used for significant (p-value <0.05) effects by comparing the least squares means of the corresponding treatment combinations. Letter groupings were generated using a 5 % level of significance for the main effects (treatment and time) and using a 1 % level of significance for temperature and turgor changes over time (treatment by hour interaction effect). For each response, the validity of model assumptions were verified by examining the residuals as described in Montgomery (2013).

Stress-responses of control and Acadian^®^-treated plants were analyzed, using the initiation of wilting at Day 3 as the initial point (hour 0). Leaf temperature kinetics was analyzed with asymptotic model (Eq. 3).

3 $$Y = \theta_{1} - \theta_{2} \exp ( - \theta_{3} X) + \varepsilon$$

where *Y* is the dependent variable (temperature), *X* is the independent variable (time), *θ*_1_, *θ*_2_, *θ*_3_ are model parameters, and the error term ε is assumed to have a normal distribution with constant variance.

The parameters of this non-linear regression model were estimated iteratively using the NLIN Procedure of SAS (SAS Institute Inc. 2014) and the models were checked for adequacy (Bates and Watts 2007). Validity of the normality, constant variance and independence assumptions on the error terms were verified by examining the residuals (Bates and Watts 2007). Relationships between leaf temperature and turgor were explored for one experiment on Day 3 by using Lowess Smoother function of Minitab (Cleveland 1979).

## Results

**Fig. 2:**
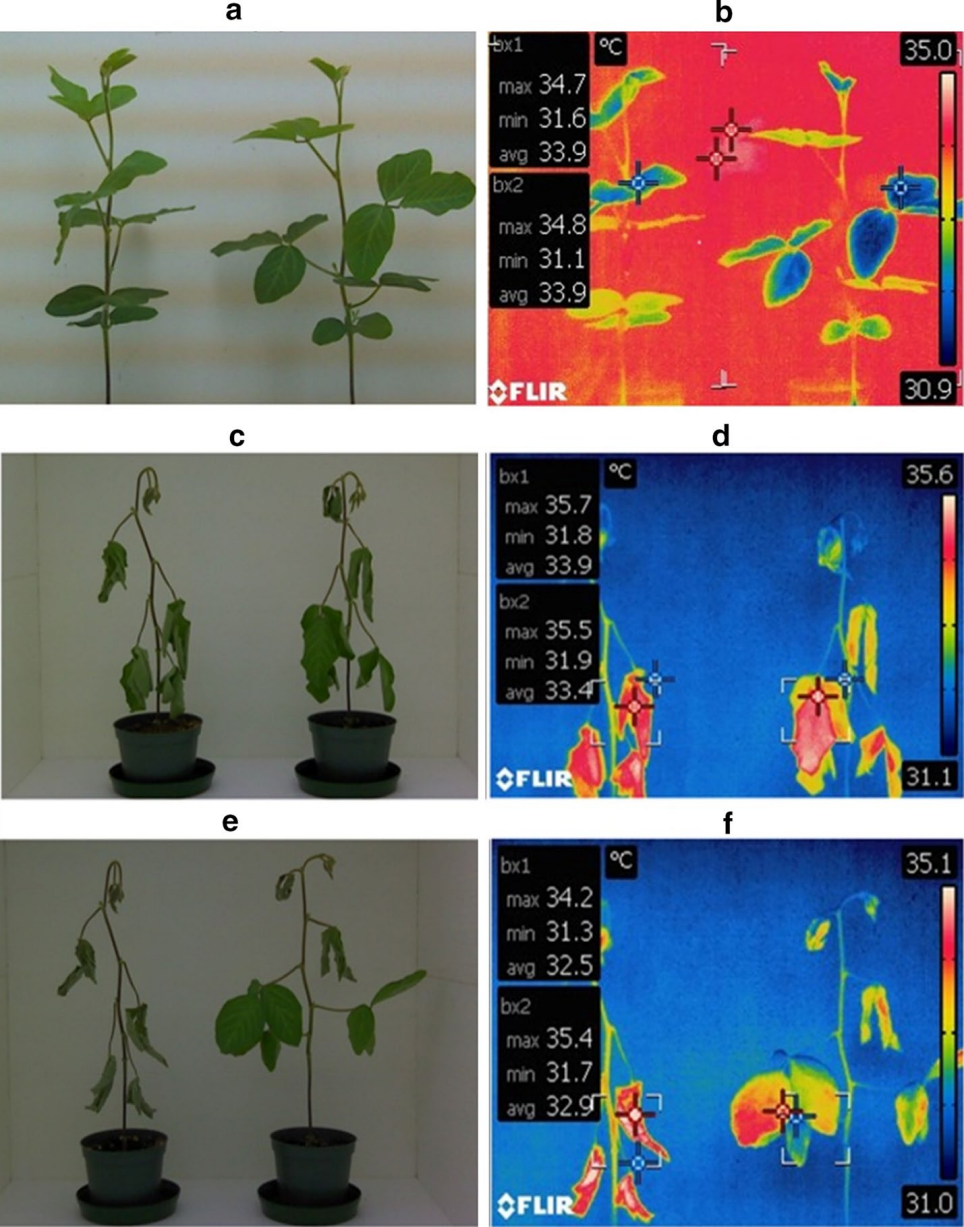
Visible (**a**, **c**, **e**) and thermal (**b**, **d**, **f**) images of soybean plants (Acadian^®^-treated—*right*, control—*left*), taken by FLIR T-440 camera in environmental chamber Conviron, air temperature 30 °C, relative humidity 60 % on Day 1 (**a**, **b**), Day 4 (**c**, **d**) and Day 5 (**e**, **f**)

Typical appearance of control and Acadian^®^-treated soybean plants over the 5-day stress-recovery experiment is shown in Fig. 2.

During the first 2 days of the experiment there was no statistically significant difference in leaf temperature and turgor between the treated and control plants (a, b). The difference between treated and control plants became significant on Day 3. After 5–6 h of light exposure control plants rapidly wilted, while treated plants still maintained turgor. After 24 h of stress, on Day 4, the turgor of Acadian^®^-treated plants was slightly higher, than the control (c, d). In the afternoon of Day 4, the moisture content in the pots was ~10–13 %. Re-watering on Day 5 showed significant visible difference between treatments. Within 2–3 h after re-watering, all Acadian^®^-treated plants recovered, while control plants were not able to fully recover (e, f). Consistent positive effect of seaweed treatment was verified in multiple experiments.

**Fig. 3:**
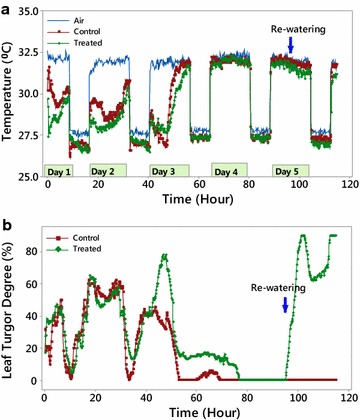
An example of leaf temperature (**a**) and turgor (**b**) of Acadian^®^-treated plants versus control over a 5-day period. Experiment was carried out in Conviron chamber in Cornwallis with one control and one treated plant

The changes of leaf temperature and leaf angle over 5-day stress-recovery experiment are presented in Fig. 3. Initial rapid decrease of leaf temperature on Day 1 (Fig. 3a) could be explained as a response to initial soil saturation. On Day 1 and at the beginning of Day 2 the temperature of the plant leaves was constant, at around 28–29 °C, which indicated that water was still available to the plants. The leaf temperature was 3.0–3.5 °C below air temperature, which corresponded to normal transpiration. Towards the end of Day 2 the temperature of the both plants increased, which indicated partial closure of the stomata due to water becoming the limiting resource. The difference between air and leaf temperatures decreased to 2.0–1.5 °C, which could be interpreted as a decrease in stomatal conductance. These changes in leaf temperature were accompanied by corresponding changes in leaf angle (Fig. 3b). At night, the leaves were in low position, increasing to 50°–60° during the day, which indicated an availability of water. During the daytime, the leaf temperature of the control plant was higher, which suggests better cooling ability of Acadian^®^-treated plants; however, visible images did not show any significant difference between the Acadian^®^-treated and control plants.

From Fig. 3a, three distinct stages of plant response were recognized:

Stage 1: (Day 3): At the beginning of the day, there were no visual differences between plants. During the first 4–5 h of the daytime period, the temperature of both control and Acadian^®^-treated plants significantly decreased (i.e., the leaf-air temperature increased to 4.5–5.0 °C), probably due to increased stomatal conductance. It is interesting, that the leaf angle of Acadian^®^-treated plants demonstrated a short-term increase, as compared to a gradual drop of turgor pressure as observed in the control (Fig. 3b). Since leaf turgor is a good indicator of stomatal opening (Jones et al. 2009), it could be postulated that the treated plants were able to control their stomatal apertures under water stress conditions.

On Day 3 after 5–6 h of light exposure the water stress caused wilting of control plants. The temperature of the control plants increased from 28.0 to 32.0 °C, approaching equilibrium with the air temperature (see Fig. 3a). This was accompanied by a rapid decrease in leaf angle (see Fig. 3b), which could be interpreted as a loss of control over stomatal opening during the water stress conditions. In contrast, the temperature of the Acadian^®^-treated plants increased slowly with a slow decrease of leaf angle, which could be explained by better stomatal control.

Stage 2 (Day 4): No differences in leaf temperature, but small differences between the turgor of the control and Acadian^®^-treated plants were observed. Turgor pressure of Acadian^®^-treated plants was slightly higher, than control (Fig. 3b). We could assume that Acadian^®^-treated plants were able to maintain functionality of stomata openings in stress conditions.

Stage 3: (Day 5): After a day with higher than normal leaf temperature, both plants were re-watered. The visual difference between treatments was apparent. Within 2–3 h after re-watering, all Acadian^®^-treated plants recovered turgor, while control plants were not able to fully recover (Fig. 3b). Further thermal imaging showed that the Acadian^®^-treated plants were able to gradually reduce their leaf surface temperature by 2–3 °C below ambient temperature, indicating a return to normal transpiration. In contrast, the leaf surface temperature of the control plants did not change, remaining in equilibrium with the ambient temperature. This can be explained by the fact that the control plants lost their ability to transport water and recover turgor in some leaves. The observed difference allowed us to conclude that Acadian^®^-treated plants were better able to withstand the types of drought stress as imposed under the experimental conditions.

**Fig. 4:**
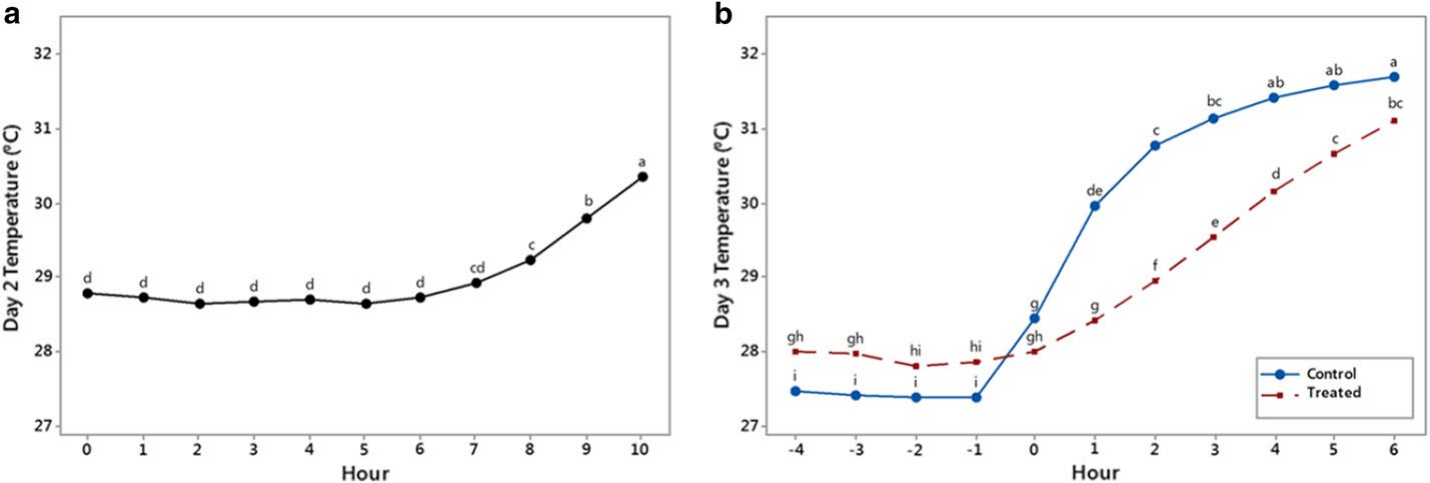
Mean temperature on Day 2 (**a**), and mean temperature of the control and Acadian^®^-treated plants on Day 3 (**b**: stress). Within each day, means sharing the *same letter* are not significantly different

To understand physiological aspects of the response, data from three different experiments were analyzed using the treatment (Acadian^®^-treated and control) as the primary factor of interest, experiment as a blocking factor (3 blocks), and time (10 h of daytime period) as the repeated measures factor. The statistical analyses of the data collected from the plant responses on Day 2, 3 and 4 are presented in Table 1 and Fig. 4.

**Table 1:** ANOVA p-values for the main and interaction effects of block, treatment and time on Day 2 temperature (°C), Day 3 temperature (°C), Day 3 turgor (%), and Day 4 temperature (°C)

Source of variation	Day 2 temperature	Day 3 temperature	Day 3 turgor	Day 4 temperature
Block	0.707	0.349	0.429	0.720
Treatment	0.884	0.017	*0.062*	0.859
Time	*0.001*	0.001	*0.001*	0.799
Treatment × time	0.992	*0.001*	0.782	0.947

Significant effects that required multiple means comparison are shown in italics

The results showed that the differences among the 3 Blocks (experiments) were not significant, which indicated a consistent stress-response in all experiments. In Day 1 the difference in leaf temperature and turgor between the two treatments was not significant (not shown in the Table). In Day 2 the difference between the leaf temperatures from the two treatments was not significant for the first 6 h, but become significant for the last 4 h of daytime period. Figure 4a shows significant increase of leaf surface temperature observed during the last 4 h of the daytime period. For Day 3 we observed a significant interaction effect of treatment and time (hour) on leaf temperature, which indicated that the plant responses to the applied stress between the Acadian^®^-treated and control plants changed over the period of observation (Fig. 4b). As a result, we separated the time series data in two clusters (−4 to 0 h, before wilting) and (0 to 6 h, after wilting), which allowed us to distinguish two phases of the stress response.

Analysis of the leaf turgor stress-response on the Day 3 showed significant difference between the Acadian^®^-treated and control plants. The turgor of treated plants was significantly higher (43.52 %) than control (23.31 %).

**Fig. 5:**
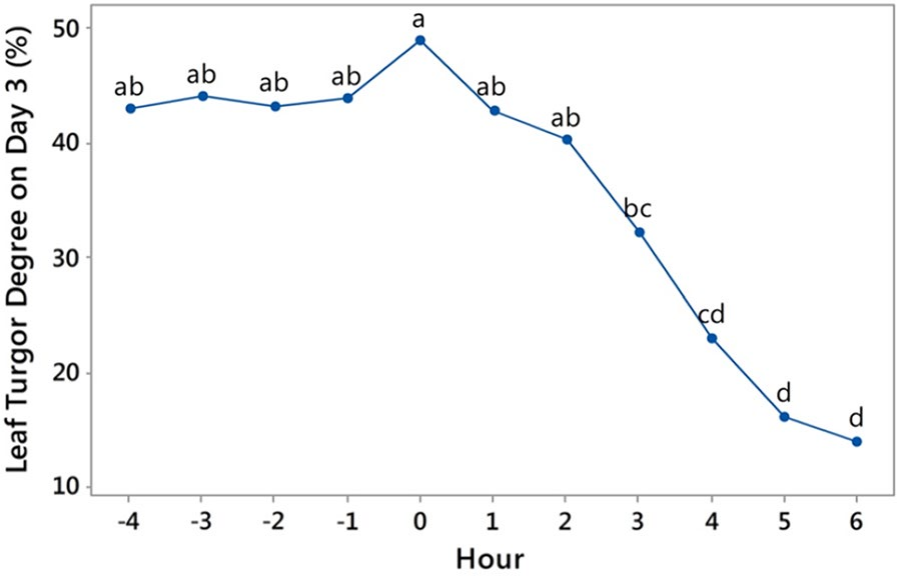
Main effect plot of time on Day 3 leaf turgor (%) showing trends of the mean values for 11 h. Means sharing the *same letter* are not significantly different

As shown in Table 1, the interaction between Treatment and Time on the leaf turgor, calculated on Day 3 was not significant (p = 0.782), which implies that the difference between average turgor of treated and control plants was consistent during the 10 h. Since the interaction effect was not significant for each hour, the mean value shown in Fig. 5 is average of the values obtained from treated and control treatments.

Figure 5 shows that leaf turgor increased marginally just before time point “0”, which indicated the “critical point of stress”. It is possible that this is an indication of a protective stress-response, such as short-term closing of the stomata in response to drought. However, further drought stress resulted in a gradual decrease of turgor.

### The difference in early stress-response

**Fig. 6:**
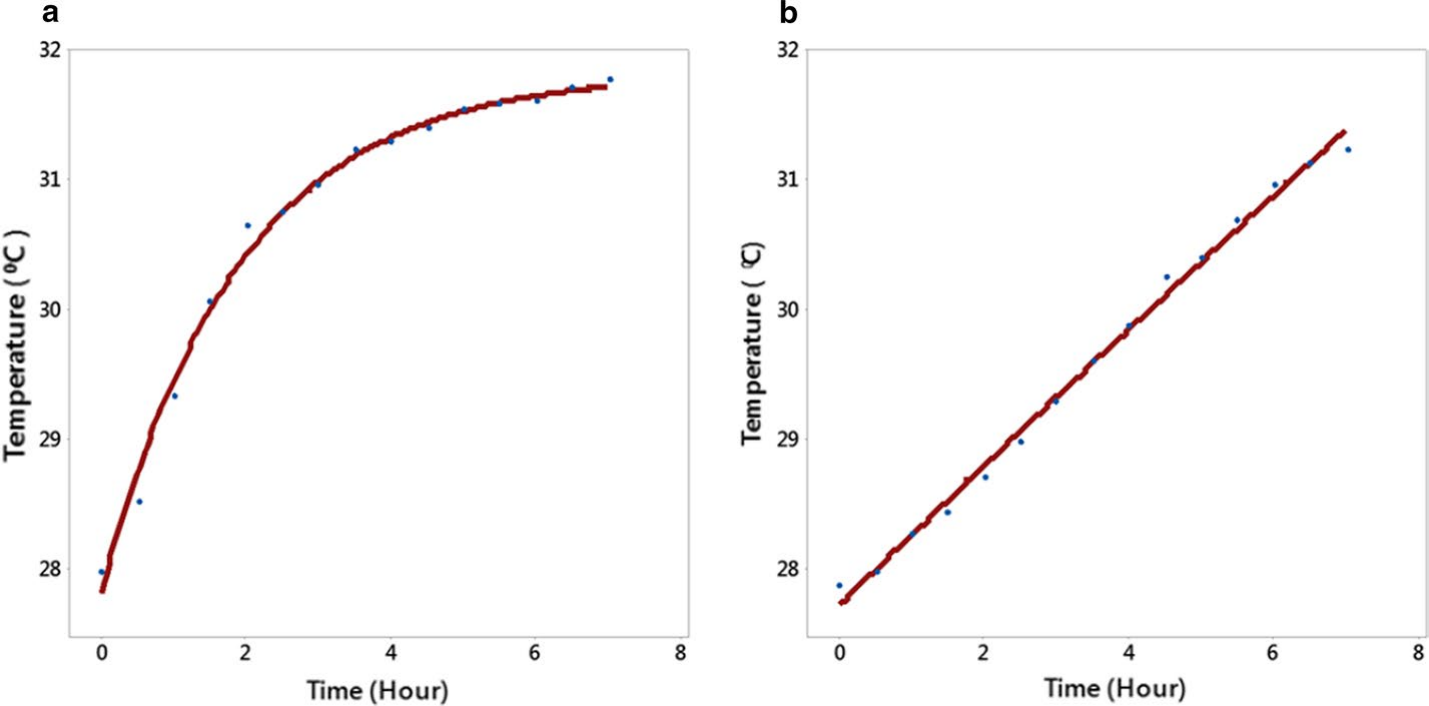
Leaf surface temperature of the control (*left*) and Acadian^®^-treated (*right*) related to the time (8 h after wilting). Each experimental point is the average of three replications. **a** Temperature (control) = 31.83 − 4.0 × exp(−0.523 × time), **b** temperature (treated) = 82.81 − 55.1 × exp(−0.0098 × time)

To determine the difference in stress-response of the Acadian^®^-treated plants versus control, the leaf temperature kinetics at the onset of physiological stress were analyzed with regression analysis. Fitted asymptotic models for the control and Acadian^®^-treated plants with 30 min increments are presented in Fig. 6. The leaf temperature of control plants followed asymptotic behavior, whereas Acadian^®^-treated plants demonstrated linear changes.

A plausible explanation for these observations could be provided from thermodynamic principles. Water evapotranspiration from stomata provides heat balance between the plant leaf and the environment. The asymptotic model usually describes a transient, first-order process of equilibration with ambient conditions. From Fig. 6a, it follows that the leaf temperature of the control plants asymptotically reached equilibrium with the ambient temperature. The equilibrium temperature, predicted by the model, was 31.8 °C, which is close to the ambient air temperature (32 °C). The observed passive response could be interpreted as a failure of plant evapotranspiration.

In contrast, the almost linear response of the Acadian^®^-treated plants showed that their status was far from thermal equilibrium. This type of response could be interpreted as an active control of the plant thermal regime due to Acadian^®^ treatment.

### Correlations between leaf turgor and temperature

**Fig. 7:**
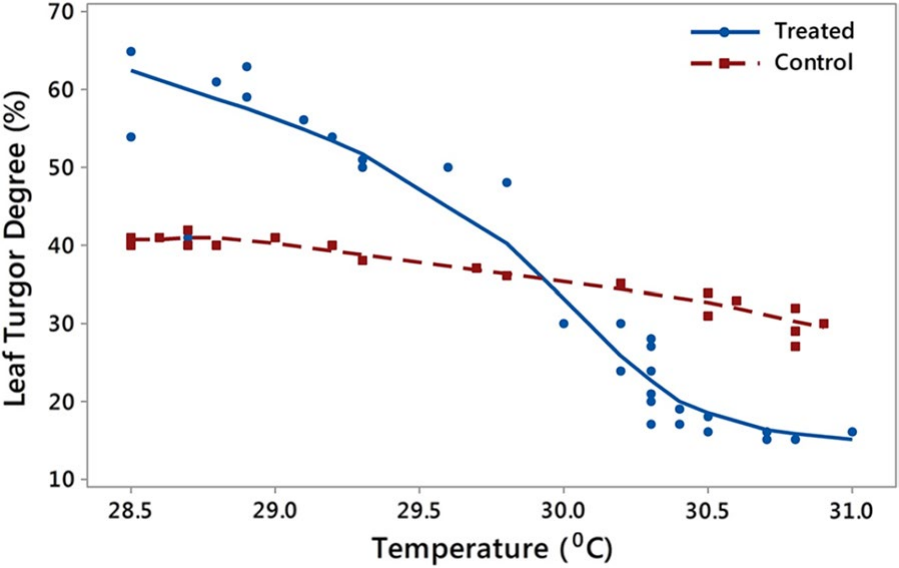
Leaf turgor-leaf temperature relationship for Acadian^®^-treated (*solid*) and control (*broken*) plotted for Day 3

To further explore if there was any relationship between turgor and leaf temperature, a correlation analysis was carried out. To understand if there were any differences in the stress response between the Acadian^®^-treated and control plants, leaf surface temperature-turgor data in the range of 28.5–31.0 °C for Day 3 were plotted (Fig. 7).

It was concluded that for both treatments, increasing leaf surface temperature was accompanied by corresponding decreased turgor pressure (i.e., a negative correlation). However, the difference in slope was significant (p < 0.05). Acadian^®^-treated plants demonstrated higher sensitivity to changes of leaf temperature (i.e., better ability to regulate turgor with critical temperature increase). These results suggested that Acadian^®^-treated plants adjusted turgor with respect to leaf temperature changes, while control plants were trying to maintain constant turgor over the range of leaf temperatures.

## Discussion

Results of our study demonstrated the positive effect of Acadian^®^ seaweed extract on stress resistance and recovery of soybean plants. The general tendency of increasing leaf temperature during the daytime period in Day 1 and 2 was observed for both Acadian^®^-treated and control plants. The observation could be attributed to the physiological phenomenon of midday depression of photosynthesis, caused by a decrease of stomatal conductance at high light intensity (Tenhunen et al. 1987). This increase was even more evident on Day 2 (Fig. 3a), which indicated limited water availability and its effect on partial stomata closure. Based on previous research (Wally et al. 2013), we could assume that stomata control in the first two days of drought stress was accompanied with the synthesis of endogenous ABA, especially in Acadian^®^-treated plants. This assumption, however, requires careful experimental verification.

On Day 3, the combination of water deficit with high irradiation significantly changed the physiological response of the leaves. In the first 7 h of the daytime period the leaf temperature decreased from 28.5 to 27.5 °C in the treated, and even to 26.5 °C in the control plants, which indicated progressive increase of transpiration. This phenomenon could be explained as a rapid initial stomata opening before the long-term closure in conditions of water deficit, known as “Iwanov” effect (Iwanov 1928). Since soil water was not available at this point of the drought stress, we can speculate that plants gave up some intracellular water. Consequences of this were dramatic and different for the control as compared to Acadian^®^-treated plants. The next changes of leaf temperature showed different patterns: the temperature of the control leaves asymptotically approached thermal equilibrium (Fig. 6), while the Acadian^®^-treated plants demonstrated a gradual increase of leaf temperature, typical for ABA-controlled stomatal closure. It is obvious that the Acadian^®^ treatment improved the physiological ability to withstand the water stress. Three stages of plant stress-response, identified in our experiments, could be attributed to three stages of General Adaptation Syndrome (GAS): alarm, resistance and exhaustion/survival (Selye 1950). There is still discussion whether or not GAS concept is applicable to plant physiology (Leshem and Kuiper 1996). Regardless of the answer, our results indicate that treatment with Acadian^®^ seaweed extract provided successful adaptation and survival of treated plants as compared to control. However, the biochemical mechanism of the action of a commercial extract of *Ascophyllum nodosum* remain unclear and require further investigation.

## Conclusions

Thermal imaging of leaf temperature can be used as an indicator of stomatal closure in response to soil water deficit. Turgor and leaf temperature changes provide vital information to enhance the scientific understanding of plant physiological stress. The early-stage response of plants to drought stress was modified by prior treatment with Acadian^®^ seaweed extract, which resulted in better adaptation and survival of Acadian^®^-treated plants.
